# Factors influencing the health-related quality of life in Korean menopausal women: a cross-sectional study based on the theory of unpleasant symptoms

**DOI:** 10.4069/kjwhn.2022.05.29

**Published:** 2022-06-29

**Authors:** Ji-Hyun Kang, Moon-Jeong Kim

**Affiliations:** Department of Nursing, Pukyong National University, Busan, Korea

**Keywords:** Middle aged, Menopause, Psychological distress, Quality of life, Social support

## Abstract

**Purpose:**

Based on the theory of unpleasant symptoms (TOUS), this study aimed to examine the direct effect of antecedent factors on health-related quality of life (HRQoL) and its indirect effect via symptoms in Korean women during the late menopausal transition (MT) and early postmenopause.

**Methods:**

This cross-sectional survey employed a descriptive correlational research design. The respondents were 152 middle-aged women 40 to 60 years with an intermenstrual interval of 60 days or more (late MT) or less than 5 years from the last menstrual period (early postmenopause). The respondents were recruited through convenience sampling in Busan, Korea, from December 1, 2020, to January 31, 2021. Based on the TOUS, self-report data were collected on perceived health status, psychological distress, social support, menopausal symptoms, and HRQoL. The collected data were analyzed using descriptive statics, independent t-test, one-way analysis of variance, Pearson’s correlation coefficient, and the Hayes’ PROCESS macro.

**Results:**

TOUS was supported on this sample (n=152) of Korean women during the late MT and early postmenopause. Perceived health status, psychological distress, and social support had significant direct relationships with HRQoL. Menopausal symptoms had significant indirect relationships between antecedent factors (perceived health status, psychological distress, and social support) and partially mediated HRQoL.

**Conclusion:**

The findings of this study indicate that menopausal symptoms play an important role as an intervening factor of HRQoL in women during the late MT and early postmenopause. Therefore, women need an integrated program that manages antecedent factors and menopausal symptoms to improve HRQoL in these menopausal stages.

## Introduction

Healthy life expectancy (HLE) is the expected number of years to live in good health, excluding periods of illness or injury [1]. Statistics Korea published the HLE estimate for people born in 2018 for the first time [2]. HLE also includes a dimension of quality of life based on the length of time a person expects to maintain a healthy lifestyle [1]. In Korea, the average life expectancy of women born in 2018 is 85.7 years, and that of women born in 2030 is 90.8 years [2], which exceeds 90 years of age for the first time in world history [3]. Given that the average HLE for women born in 2018 is only 64.9 years, women born in 2018 will live with disease and disability for about 21 years [2]. Women live on average 6 years longer than men, but HLE is estimated to be 0.9 years longer [2]. Therefore, since women may have worse health from around the age of 65 than before, more active health management is needed from middle age onwards.

Health-related quality of life (HRQoL) is a broad concept that includes physical, mental, emotional, and social functioning [4]. Many studies have reported the following factors affecting HRQoL in middle-aged women. General characteristics such as age, economic status, and level of education [5]; physiological factors such as obesity, menopausal conditions, and comorbidities [5]; psychological factors such as perceived health status, stress [6], and depression [7]; social factors such as social support and occupation [8,9].

Menopause is a spontaneous, unavoidable event that every woman will experience. Therefore menopause is considered the most crucial factor in the well-being of middle-aged women [5,7]. During the menopausal period, women experience menopausal symptoms such as hot flushes, night sweats, sleep problems, chilling, mood changes, vaginal dryness, and arthralgia [5]. The menopause stage is classified by the STRAW (Stages of Reproductive Aging Workshop) staging system, widely considered the standard for menopause [10]. According to STRAW, the late menopausal transition (MT) occurs when an intermenstrual interval equal to or greater than two skipped cycles or more than 60 days. Early postmenopause is 5 years after the final menstrual period. A prospective, longitudinal study in Korea with 2,204 women aged between 44 to 56 years old reported that women in late MT and postmenopause have more severe menopausal symptoms than the other stages of menopause [5]. Therefore, menopausal symptoms may affect HRQoL of women during the late MT and early postmenopause.

Symptom management is paramount in nursing practice and emerges as an essential focus of nursing science [11]. Nurses can provide symptom-focused care, including measuring symptoms, assessing factors that affect symptoms, preventing worsening of symptoms, and managing to alleviate symptoms [12]. Symptoms are the central focus of the theory of unpleasant symptoms (TOUS), a mid-range theory developed by Lenz et al. [11]. TOUS consists of three main concepts; antecedent factors, symptoms, and performance. Physiological, psychological, and situational factors are antecedent factors that directly and indirectly influence performance, and symptoms are proposed to mediate this relationship [12].

Late MT and early postmenopausal women experience more severe menopausal symptoms, which can change their HRQoL [5]. Therefore, this study aims to explain HRQoL of Late MT and early postmenopausal women by applying TOUS, focusing on menopausal symptoms. This study may provide practical implications for developing strategies and interventions to improve Korean women’s HRQoL.

Based on the TOUS, this study aimed to examine the effect of perceived health status, psychological distress, social support, and menopausal symptoms on HRQoL and the mediating effects of menopausal symptoms in Korean women during the late MT and early postmenopause. The specific objectives are as follows: (1) to investigate the differences in HRQoL by general characteristics and health-related characteristics; (2) to examine the levels of perceived health status, psychological distress, social support, menopausal symptoms, and HRQoL and to analyze the correlation between these variables; (3) to investigate the direct effect of antecedent factors on HRQoL and indirect effects through menopausal symptoms; and (4) to test the statistical significance of indirect effects of menopausal symptoms.

## Methods

Ethics statement: This study was approved by the Institutional Review Board of Pukyong National University (1041386-202010-HR-60-02). Written informed consent was obtained from all participants.

### Study design

**Figure 1.**
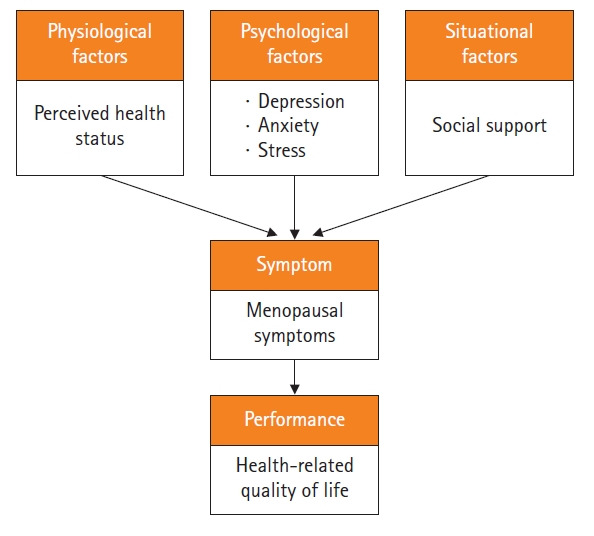
Framework of the present study based on the Theory of Unpleasant Symptoms.

This cross-sectional survey employed a descriptive correlational design to examine the direct effect of perceived health status, psychological distress, and social support on HRQoL, and indirect effects through menopausal symptoms in late MT and early postmenopausal women. Based on TOUS and previous studies [5-9], the theoretical framework of this study was as follows: antecedent factors (perceived health status as a physiological factor, psychological distress as a psychological factor, and social support as a situational factor); symptoms (menopausal symptoms); and performance (HRQoL). Also, the theoretical framework was developed by applying the pathway in which the antecedent factors affect the symptoms, and the symptoms affect performance (Figure 1). This study report followed the STROBE (Strengthening the Reporting of Observational Studies in Epidemiology) reporting guidelines (https://www.strobe-statement.org/).

### Participants

Participants were recruited via convenience sampling in Busan, Korea. The inclusion criteria were as follows: (1) middle-aged women, 40 to 60 years; and (2) intermenstrual interval of 60 days or more (late MT) or less than 5 years from the last menstrual period (early postmenopause) [10]. The exclusion criteria were as follows: (1) women on hormonal therapy; and (2) those who had experienced artificial menopause (due to hysterectomy, bilateral ovarian removal, or chemotherapy).

### Sample size

Using the G-power 3.1.2 program, the minimum sample size for multiple regression analysis was calculated (significance level, .05; power, .80; effect size, .15 based on previous studies [6]; 14 predictors including 10 items of general characteristics and health-related characteristics, and four main variables). The minimum sample size required 143 respondents. The questionnaire was distributed to 160 people considering a dropout rate of 15%. Finally, 152 completed questionnaires were statistically analyzed.

### Instruments

Consent for all measurements in this study was obtained from both original developers and Korean version translators via e-mail.

#### Physiological factor: perceived health status

The Perceived Health Status Scale developed by Speake et al. [13] and translated into Korean [14] and validated [15] was used in this study. This scale consists of three items that measured current health status, degree of daily-life disturbances, and health status compared to the other people in the same age group. Items are rated on a 5-point Likert scale from 1 (very bad) to 5 (very good) and higher summed scores (range, 3–15) indicate good perceived health status. Cronbach’s alpha of the Korean version was .82 [14] and .80 in this study.

#### Psychological factors: psychological distress

The Depression, Anxiety, and Stress Scale (DASS-21) was developed by Lovibond and Lovibond [16] and was translated into Korean and validated by Cha et al. [17]. This scale consists of 21 items in three domains (depression, anxiety, and stress). A 4-point Likert scale (0, did not apply to me at all to 3, applied to me very much) is used and higher summed scores (range, 0–63) indicate high psychological distress. Domain scores can range from 0–21 for depression, 0–21 for anxiety, and 0–21 for stress. Cronbach’s alpha was .94 in Cha et al. [17] and .91 in this study.

#### Social factors: social support

Social Support Scale was developed and validated by Park [18]. This scale consists of 25 items in four domains (emotional support, evaluational support, material support, and informational support). Items are rated on a 5-point Likert scale from 1 (strongly disagree) to 5 (strongly agree) and higher summed scores (range, 25–125) indicate high social support. Cronbach’s alpha was .95 in Park [18] and .71 in this study.

#### Symptoms: menopausal symptoms

The Menopause Rating Scale (MRS) developed by Heinemann [19] and translated into Korean and validated [20] was used to measure menopausal symptoms. This scale consists of 11 items in three domains (somatic symptoms, psychological symptoms, and urogenital symptoms). Scores are summed (range, 0–44) with each item rated on a 5-point Likert scale (0, no complaints to 4, severe symptoms). Scores are interpreted as follows: 0 to 4 (no/little complaints), 5 to 7 (mild symptoms), 8 to 15 (moderate), and 16 to 44 (severe symptoms). Cronbach’s alpha was .86 in Heinemann [19] and .80 in this study.

#### Performance: health-related quality of life

The Korean version [21] of the SmithKline Beecham Quality of Life Scale [4] was used to measure HRQoL. This scale consists of 23 items in five domains (competence, psychological well-being, physical well-being, stability, and activity). Items are rated on a 10-point Likert scale from 1 to 10 and higher summed scores (range, 23–230) indicate better HRQoL. Cronbach’s alpha of the Korean version was .89 [21] and .80 in this study.

#### General characteristics and health-related characteristics

General characteristics included age, education, religion, occupation, spouse, and economic state. Health-related characteristics included body mass index, menopausal stage, parity, and comorbidity.

### Data collection

This study was conducted in Busan, Korea, from December 1, 2020 to January 31, 2021. Following ethical approval, the researcher visited the representatives of social welfare centers, gymnasiums, churches, and hospitals and received permission to post public information about this study on the wall. Interested participants who contacted the research team were provided with the questionnaire set and return envelope after informed consent. After completion, respondents put the questionnaires in an envelope, sealed them, and placed them in a storage box at the site. The average time for respondents to complete the questionnaire was 15 to 20 minutes. A gift worth 4 thousand Korean won (approximately 3 US dollars) was given to respondents who completed the questionnaire.

### Data analyses

The collected data were analyzed using IBM SPSS ver. 25.0 (IBM Corp., Armonk, NY, USA). The general characteristics and health-related characteristics were analyzed with descriptive statistics such as frequency, percentage, and mean. The differences in HRQoL according to general characteristics and health-related characteristics were analyzed by independent t-test and one-way analysis of variance. Pearson’s correlation analysis was used to test relationships among perceived health status, psychological distress, social support, menopausal symptoms, and HRQoL.

Before the analyses, multicollinearity assumptions were examined through tolerance and variance inflation factor (VIF). Tolerance ranged from 0.69 to 0.80, VIF ranged from 1.26 to 1.46, indicating an absence of multicollinearity, thus satisfying basic regression assumptions. The Durbin-Watson statistic was 1.91, indicating no autocorrelation in residuals.

The mediating effect of menopausal symptoms in the relationship between antecedent factors (perceived health status, psychological distress, and social support) and HRQoL was analyzed using simple mediation (model 4) analysis using the PROCESS macro for SPSS. The Baron and Kenny method and Sobel test are traditional approaches to mediation analysis, and have the issue of normality of the indirect effect [22]. In these methods, the distribution of indirect effect tends to be valid only in a large enough sample size. Hayes’ bootstrapping [22] is a resampling method to test a confidence interval (CI) for the indirect effect and has an advantage over these two methods. The number of resampling bootstraps was chosen to be 5,000 times to verify the significance of the indirect effect of menopausal symptoms.

**Figure 2.**
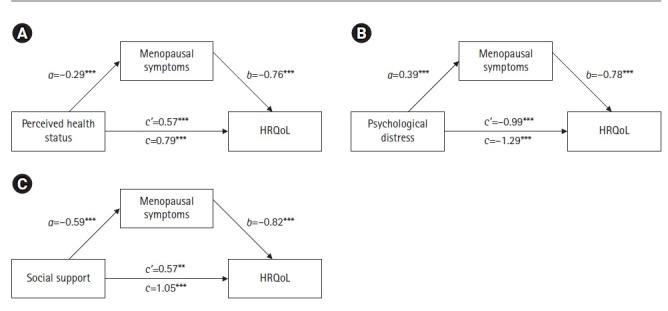
Mediating effect of menopausal symptoms in the relationship between perceived health status, psychological distress, social support, and health-related quality of life (HRQoL) (N=152). All paths (*a, b, c*, and *c’*) are coefficients representing unstandardized regression weights. *a, b, c’*=direct effects; *c*=total effect. *p<.05, **p<.01, ***p<.001.

The path from independent variables to mediator variable is called *a*; the path from mediator variable to dependent variable is called *b*; and the path from independent variables to dependent variable is called *c’* (“direct effect”) (Figure 2). The indirect effect of independent variables on dependent variable through mediator variable is obtained by multiplying *a* and *b*. The total effect, *c*, is the sum of the direct effect and the indirect effect. The indirect effect (*c-c’*=*ab, ab* was also known as “mediation effect”) is indicated by a statistically significant difference between *c* and *c’*. The indirect effect was statistically significant if the 95% bias-corrected CIs for the indirect effect did not include zero [22].

## Results

### Differences in health-related quality of life by respondent characteristics

The highest proportion of respondents is aged 55 to 60 years (46.7%). The majority of respondents attained a high school level of education (67.1%) and identified with a religion (84.9%). Most were also employed (73.0%) and had spouses (88.2%). More than half responded that their financial status was middle-range (59.9%). Roughly half were normal weight (46.7%) and 67.1% were early postmenopause. The majority of respondents had a parity of 1 or 2 times and 59.9% had no comorbidity. Respondents with comorbidity had a lower HRQoL than those without (t=–2.83, *p*<.001) (Table 1).

### Levels of perceived health status, psychological distress, social support, menopausal symptoms, and health-related quality of life

HRQoL was of moderate level at 131.17±19.51, perceived health status (9.34±1.95), and psychological distress (33.88±9.05) were also at moderate levels. The three domains of psychological distress were all at moderate levels: depression (9.59±2.82,), anxiety (9.78±3.71), and stress (14.51±2.52). Social support was also of moderate level (65.82±10.14), whereas overall menopausal symptom scores were 22.29±4.84, indicating severe menopausal symptoms (Table 2).

### Relationships among perceived health status, psychological distress, social support, menopausal symptoms, and health-related quality of life

HRQoL showed a significantly positive correlation with perceived health status (r=.58, *p*<.001) and social support (r=.49, *p*<.001) but showed a significant negative correlation with psychological distress (r=–.62, *p*<.001) and menopausal symptoms (r=–.59, *p*<.001) (Table 3).

### Mediating effect of menopausal symptoms

Results on the direct effect of the independent variable (perceived health status, psychological distress, and social support) on the dependent variable (HRQoL) showed that perceived health status had a significant positive effect on HRQoL (B=0.57, *p*<.001) (Figure 2A). Psychological distress had a significantly negative effect on HRQoL (B=–0.99, *p*<.001) (Figure 2B), whereas social support had a significant positive effect on HRQoL (B=0.57, *p*<.001) (Figure 2C).

Next, the indirect effect of the independent variable (perceived health status, psychological distress, and social support) on the dependent variable (HRQoL) through the mediator variable (menopausal symptoms) (Figure 2) found that perceived health status had a significantly negative effect on menopausal symptoms (B=–0.29, *p*<.001), and menopausal symptoms had a significantly negative on HRQoL effect (B=–0.76, *p*<.001). Thus, perceived health status had a significant indirect effect on HRQoL that was mediated by menopausal symptoms. To investigate the significance of the indirect effect, using the bootstrapping method we identified the mediating effect of menopausal symptoms on perceived health status and HRQoL was –0.22, which was significant (95% bias-corrected bootstrap CI, 0.11 to 0.34) (Table 4).

Psychological distress had a positive effect on menopausal symptoms (B=0.39, *p*<.001), and menopausal symptoms had a significant negative effect on HRQoL (B=–0.78, *p*<.001). Thus, psychological distress had a significant indirect effect on HRQoL that was mediated by menopausal symptoms. The mediating effect of menopausal symptoms on psychological distress and HRQoL was also significant, with a value of –0.31 (95% bias-corrected bootstrap CI, –0.49 to –0.15) (Table 4).

Social support had a negative effect on menopausal symptoms (B=–0.59, *p*<.001), and menopausal symptoms had a significant negative effect on HRQoL (B=–0.82, *p*<.001) (Table 4). Thus, social support had a significant indirect effect on HRQoL that was mediated by menopausal symptoms. The mediating effect of menopausal symptoms on social support and HRQoL was 0.49, which was significant (95% bias-corrected bootstrap CI, 0.27 to 0.72) (Table 4). In conclusion, the independent variables had both direct and indirect effects on the dependent variable. Also, the indirect effects of the independent variable on the dependent variable through the mediation variable were smaller than the direct effects of the independent variable on the dependent variable (Table 3). These results indicate that menopausal symptoms partially mediated the effects of perceived health status, psychological distress, and social support on HRQoL.

## Discussion

This study investigated the effect of antecedent factors (perceived health status, psychological distress, and social support) and symptoms (menopausal symptoms) on performance (HRQoL) based on TOUS. The mediating effect of menopausal symptoms on the relationship between antecedent factors and performance was also investigated.

This study found a significant difference in HRQoL according to the comorbidities. It was consistent with previous studies in which those with fewer comorbidities had a higher HRQoL [7] and those who had no comorbidity had higher HRQoL [8]. Discomfort or stress caused by comorbidity may affect the low HRQoL. Nurses need to assess the presence of comorbidities to improve the HRQoL. The average HRQoL score in this study (131.17) was lower than the women aged 40 to 60 years in the study of Jung and Chun [9]. It might be because the proportion of respondents with comorbidities in this study was higher (40.1%) than in Jung and Chun’s study (25.9%). On the other hand, this score is higher than the HRQoL score reported for older Korean women living alone [23]. These results may be due to the presence of spouse and age as some studies reported that quality of life was lower among those with no partner [8,23] and older age [5,8,23].

As proposed by the TOUS, this study supported that antecedent factors had a significant effect on performance; perceived health status, psychological distress, and social support showed significant direct effects on HRQoL. Perceived health status having a direct relationship with HRQoL support a previous study that showed perceived health status strongly affected HRQoL in middle-aged women [6]. The average score of perceived health status was 9.34, slightly lower than a previous study [24]. These results appear to be due to differences in comorbidities. In the previous [24] and this study, 21.0% and 40.1% of respondents, respectively, had comorbidity. Perceived health status is closely related to objective health status and personal attitudes for evaluating health status [8]. People who perceive their health status positively tend to invest more time and effort in maintaining health-promoting behaviors [25]. Therefore, it is necessary to efforts to help women with reduced health awareness after menopause positively perceive their health status.

Psychological distress also had a direct relationship with HRQoL. Similarly, Sohn [7] and Jung and Chun [9] reported that psychological distress lowered the quality of life in middle-aged Korean women. Some studies have shown that when the brain continues to perceive stress or anxiety, it activates the body’s inflammatory response and promotes the release of cytokines [26]. Stress and anxiety also inhibit hypothalamic function, leading to fatigue, lethargy, depression, and cognitive dysfunction. Prolonged stress can have severe physical and mental health consequences, such as increasing the risk of premature aging, cytokine-induced disease, high blood pressure, angina, ischemic heart disease, and cerebral hemorrhage [27]. In addition, severe depression causes a loss of motivation and appetite, insomnia, and lethargy to decrease the quality of health [5,7]. Park and Choi [28] conducted an intensive mindfulness-based stress reduction program (8 times a week for 2.5 hours/week) for women between the ages of 40 and 59 years and found that the experimental group had significantly lower levels of stress, depression, and anger than the control group. Mindfulness meditation may be valuable as an effective mental training method that can reduce psychological distress in postmenopausal women. In this sample, 9.9% of respondents had a severe level of depression and 5.3% had a severe level of stress, which is comparable to other reports [9,24]. In contrast, 42.1% of respondents had a severe level of anxiety, but direct comparison with prior studies due to few studies using the DASS-21 in Korean women during menopause.

Social support had a direct relationship with HRQoL. The strong association between social support and menopause-related quality of life in previous research [7] supports the results of this study. This study’s average social support score was lower than a prior study [24], which may be due to age differences; i.e., the average age of respondents in this study was 54 years old, which is higher than the 47 years in the study of Jung and Oh [24]. The risk of social isolation increases as the elderly are more likely to experience stressful life events such as losing family or friends and chronic illness [7,24]. On the other hand, studies suggest that people who help others are less stressed and happier and experience positive emotions [29], also echoed in a study that middle-aged Korean women who volunteer are healthier than those who do not [30]. As such, middle-aged women entering MT can be encouraged to participate in various volunteer activities with this new perspective.

As proposed by the TOUS, this study also found that perceived health status, psychological distress, and social support had significant indirect effect on HRQoL that was mediated by menopausal symptoms, suggesting that postmenopausal women can increase HRQoL through management of menopause symptoms.

Respondents in this study were noted to have severe menopausal symptoms with an average of 22.29 points, which was higher than those of previous studies involving postmenopausal women [31], or MT and postmenopausal women [32]. These previous studies, however, did not divide postmenopausal into early or late stages. In contrast, this study only included women in the late MT and early postmenopause, i.e., groups who may be more likely to experienced greater menopausal symptoms. Therefore, this requires consideration in interpretation of findings.

If middle-aged women do not manage menopausal symptoms, the risk of chronic diseases, depression, stress, osteoporosis, and metabolic syndrome increases, increasing the overall socioeconomic burden. According to the Korean National Health Insurance [33], the cost related to menopausal disorders is rising every year from about 33.8 billion Korean won (approximately 26 million US dollars) in 2016 to approximately 53.2 billion Korean won (approximately 41 million US dollars) in 2020. This suggests that menopausal symptoms are more than an individual problem but are a significant public health issue.

In general, pharmacological and nonpharmacological therapies have been used to relieve menopausal symptoms. Menopausal hormone therapy (MHT) represents the most common treatment for symptoms of menopause [34]. MHT relieves genitourinary syndrome such as vulvovaginal atrophy, pain with intercourse, urinary incontinence, and vasomotor symptoms such as hot flushes and night sweats [5,34]. MHT can rehabilitate sexual function and marital intimacy and reduce the psychological distress of middle-aged women [35]. Thus, in middle-aged women who receive MHT, the perception of menopause and aging will be more positive than others, with lower levels of conflict within their social networks. However, controversy continues for the advantages and disadvantages of MHT. A study [36] in Malay, Indian, and China found that women using MHT had worse mobility, pain/discomfort, and anxiety/depression, respectively, compared to non- MHT users. Therefore, women who desire MHT should consider their preference, circumstance, age at initiation, menopause age, and treatment goal.

Several studies have shown the effects of natural ways to reduce the symptoms of menopause. Examples include a 16-week exercise program that improved bone mineral density and health-related fitness [37], a 15-week weight training program that reduced menopausal symptoms by half [38], and horticultural therapy for 12 sessions (twice a week) reducing menopausal symptoms [39]. Another study aimed at eliminating negative emotional and physical symptoms had a significant positive effect on menopausal symptoms and quality of life in middle-aged women [40]. Nurses could suggest effective nonpharmacological interventions to help women reluctant to take drug therapy such as MHT.

This study adds empirical support for the TOUS. A prior study applying TOUS for colon cancer patients reported symptoms from illness and surgery negatively correlated with a physical component score and a mental component score for quality of life [41]. Another using TOUS for chronic insomnia found that perceived stress, presleep arousal, and social support influenced insomnia; while insomnia affected functional health [42]. In this study, menopausal symptoms mediated HRQoL in women during MT and early postmenopausal stage during which women may experience menopausal symptoms. The findings of these studies provide support for TOUS propositions that symptoms can mediate the link between influencing factors and performance [12]. Therefore, interventions not only for perceived health status, social support, and psychological distress; but also for menopausal symptoms are very important to improve HRQoL in middle-aged women.

Although previous studies have focused on menopausal symptoms, to our knowledge, none have examined menopausal symptoms as mediating factors in the relationship between antecedent factors and HRQoL. Instead, they commonly examined menopausal symptoms as an independent variable [7,31]. Further research studies will benefit menopausal women if including menopausal symptoms as mediating factors to fully explain HRQoL in middle-aged women.

A limitation of this study is that respondents were a convenience sample of women in late MT or early postmenopause. Therefore, study findings should be applied with caution to women in other menopausal phases. Another limitation is that researchers collected data during the period when coronavirus disease 2019 (COVID-19) spread rapidly nationwide. The COVID-19 pandemic has been a traumatic event, creating significant prolonged stress for people in every aspect of their lives such as increased stress related to work, financial matters, lowered income, and lost jobs [43]. This context might have influenced their psychological distress and social support.

Despite these limitations, this study showed that menopausal symptoms partially mediated the relationship between perceived health status, psychological distress, social support, and HRQoL in late MT and early postmenopausal women and validated the TOUS theory for use in better understanding HRQoL in middle-aged women.

In conclusion, the conceptual framework of Lenz et al. [11] about TOUS provides a better understanding to improve the HRQoL of menopausal women such as a more concrete awareness of the complexity of performance and preventive and management strategies. More variables related to physical, psychological, and situational factors affecting the quality of life of postmenopausal women need to be explored in future studies and TOUS can be a helpful framework. While nurses need to focus on relieving menopausal symptoms providing integrated interventions that encompass a variety of antecedent factors and menopausal symptoms to improve the quality of life of women during late MT and early menopause stages, may require a multidisciplinary team approach.

## Figures and Tables

**Table 1.** General characteristics and health-related characteristics of participants (N=152)

Variable	Categories	n (%)	Mean±SD	t/F	*p*
General characteristics					
Age (year)	45–49	17 (11.2)	5.63±0.61	0.11	.895
	50–54	64 (42.1)	5.73±0.83		
	55–60	71 (46.7)	5.68±0.99		
Education	≤High school	102 (67.1)	5.66±0.86	2.06	.131
	University	41 (27.0)	5.65±0.91		
	≥Graduate school	9 (5.9)	6.27±0.92		
Religion	Yes	129 (84.9)	5.69±0.93	0.02	.984
	No	23 (15.1)	5.70±0.58		
Occupation	Yes	111 (73.0)	5.78±0.79	1.66	.103
	No	41 (27.0)	5.47±1.08		
Spouse	Yes	134 (88.2)	5.68±0.88	0.38	.701
	No	18 (11.8)	5.77±0.91		
Economic state	Low	25 (16.4)	5.62±0.86	1.06	.377
	Middle	91 (59.9)	5.68±0.81		
	High	36 (23.7)	5.79±1.05		
Health-related characteristics					
Body mass index (kg/m^2^)	Underweight (<18.5)	13 (8.6)	5.68±0.75	0.83	.478
	Normal weight (18.5–22.9)	71 (46.7)	5.81±0.85		
	Overweight (23.0–24.9)	37 (24.3)	5.61±0.92		
	Obese (≥25.0)	31 (20.4)	5.54±0.92		
Menopausal stage	Late menopausal transition	50 (32.9)	5.88±0.90	1.80	.074
	Early postmenopause	102 (67.1)	5.61±0.85		
Parity	0	8 (5.3)	6.05±0.69	0.85	.431
	1–2	120 (78.9)	5.66±0.90		
	≥3	24 (15.8)	5.77±0.84		
Comorbidity^†^	Yes	61 (40.1)	5.45±0.80	2.83	.004
	No	91 (59.9)	5.86±0.90		

† Hyperlipidemia (11), hypertension (9), ovarian disease (7), diabetes mellitus (5), breast cysts (5), thyroid disease (5), heart disease (3), cerebrovascular disease (3), thyroid cancer (3), arthritis (3), and others (7).

**Table 2.** Levels of perceived health status, psychological distress, social support, menopausal symptoms, and health-related quality of life (HRQoL) (N=152)

Variable	Categories	n (%) or mean ± SD	Possible range	Data range
Perceived health status		9.34±1.95	3–15	3–15
Psychological distress		33.88±9.05	0–63	
Depression		9.59±2.82	0–21	5–18
	Low (0–9)	91 (59.9)		
	Moderate (10–13)	46 (30.3)		
	Severe (≥14)	15 (9.9)		
Anxiety		9.78±3.71	0–21	3–20
	Low (0–7)	55 (36.2)		
	Moderate(8–9)	33 (21.7)		
	Severe (≥10)	64 (42.1)		
Stress		14.51±2.52	0–21	6–20
	Low (0–14)	66 (43.7)		
	Moderate (15–18)	78 (51.3)		
	Severe (≥19)	8 (5.3)		
Social support		65.82±10.14	25–125	42–103
Menopausal symptoms		22.29±4.84	0–44	9–36
HRQoL		131.17±19.51	23–230	6–177

**Table 3.** Relationships among perceived health status (PHS), psychological distress (PD), social support (SS), menopausal symptoms (MS), and health-related quality of life (HRQoL) (N=152)

Variable	r (*p*)			
	PHS	PD	SS	MS
PHS	1			
PD	–.35 (<.001)	1		
SS	.28 (<.001)	–.37 (<.001)	1	
MS	–.39 (<.001)	.31 (<.001)	–.49 (<.001)	1
HRQoL	.58 (<.001)	–.62 (<.001)	.49 (<.001)	–.59 (<.001)

**Table 4.** Mediating effect of menopausal symptoms (MS) in the relationship between perceived health status (PHS), psychological distress (PD), social support (SS), and health-related quality of life (HRQoL) (N=152)

Effect	Independent variable	→	Dependent variable	B	95% bias-corrected bootstrap CI
Direct effect (*c’*)	PHS	→	HRQoL	0.57**	0.39 to 0.76
Indirect effect (*a*)	PHS	→	MS	–0.29***	–0.39 to –0.18
Indirect effect (*b*)	MS	→	HRQoL	–0.76***	–1.01 to –0.51
Indirect effect (*ab*)	PHS	→ MS →	HRQoL	0.22	0.11 to 0.34
Total effect (*c’+ab*)				0.79***	
Direct effect (*c’*)	PD	→	HRQoL	–0.99***	–1.30 to –0.70
Indirect effect (*a*)	PD	→	MS	0.39***	0.21 to 0.57
Indirect effect (*b*)	MS	→	HRQoL	–0.78***	–1.02 to –0.57
Indirect effect (*ab*)	PD	→ MS →	HRQoL	–0.31	–0.49 to –0.15
Total effect (*c’*+*ab*)				–1.29***	
Direct effect (*c’*)	SS	→	HRQoL	0.57**	0.30 to 0.85
Indirect effect (*a*)	SS	→	MS	–0.59***	–0.74 to –0.39
Indirect effect (*b*)	MS	→	HRQoL	–0.82***	–1.11 to –0.53
Indirect effect (*ab*)	SS	→ MS →	HRQoL	0.49	0.27 to 0.72
Total effect (*c*’+*ab*)				1.05***	

** *p*<.01,

*** *p*<.001.

→: Causal relationship between independent and dependent variable.
